# Author Correction: Generalizable deep learning model for early Alzheimer’s disease detection from structural MRIs

**DOI:** 10.1038/s41598-023-43726-2

**Published:** 2023-10-02

**Authors:** Sheng Liu, Arjun V. Masurkar, Henry Rusinek, Jingyun Chen, Ben Zhang, Weicheng Zhu, Carlos Fernandez-Granda, Narges Razavian

**Affiliations:** 1 Center for Data Science, NYU, 60 Fifth Avenue, 5th Floor, New York, NY 10011 USA; 2 Center for Cognitive Neurology, Department of Neurology, NYU Grossman School of Medicine, 60 Fifth Avenue, 5th Floor, New York, NY 10011 USA; 3 Neuroscience Institute, NYU Grossman School of Medicine, 145 E 32nd St #2, New York, NY 10016 USA; 4 Department of Radiology, NYU Grossman School of Medicine, 660 First Avenue, New York, NY 10016 USA; 5 Department of Psychiatry, NYU Grossman School of Medicine, 227 East 30th St, 6th Floor, New York, NY 10016 USA; 6 Courant Institute of Mathematical Sciences, NYU, 251 Mercer St # 801, New York, NY 10012 USA; 7 Department of Population Health, NYU Grossman School of Medicine, 227 East 30th street 639, New York, NY 10016 USA

Correction to: *Scientific Reports* 10.1038/s41598-022-20674-x, published online 17 October 2022

The original version of this Article contained an error in the Acknowledgements section.

“SL was partially supported by NSF DMS-2009752, NSF NRT-HDR-1922658, and Leon Lowenstein Foundation.”

now reads:

“SL was supported by Alzheimer’s Association grant AARG-NTF-21-848627, and received partial support from NSF DMS-2009752, NSF NRT-HDR-1922658, and the Leon Lowenstein Foundation.”

The original Article has been corrected.

