# Supplementary material for: Generalizable deep learning model for early Alzheimer’s disease detection from structural MRIs
Source: Sci Rep. 2022 Oct 17;12:17106. doi: 10.1038/s41598-022-20674-x (PMC9576679; doi:10.1038/s41598-022-20674-x)
Supplement: Supplementary file 1 — Supplementary Information. [file 41598_2022_20674_MOESM1_ESM.docx]

# **Supplementary material**

The data used in this study consists of imaging and diagnosis data from Alzheimer’s Disease Neuroimaging Initiative (ADNI) and National Alzheimer’s Coordinating Center (NACC). The scans from ADNI were acquired in digital imaging and communications in medicine (DICOM) format using Siemens (49.23%), GE (29.74%), and Philips (21.03%) scanners (details regarding the ADNI MRI data acquisition protocol can be found on ADNI’s official webpage:

<http://adni.loni.usc.edu/methods/documents/mri-protocols/>. For NACC dataset, there are no harmonized protocols, nor have the images been quality controlled or curated as it is a collection of scans from several AD centers, which reflects the real-world scenario.  (A set of proposed imaging standards is available through the NACC website <https://naccdata.org/>).

More detailed image acquisition protocols for ADNI and NACC are shown in the Supplementary Table S0. Both of these two datasets were from large-scale multicenter studies, in which subject inclusion criteria and/or image acquisition protocols can vary by study center, leading to potential difference in the scan and diagnosis rating.

Supplementary Table S0: Image acquisition protocols of two cohorts: ADNI and NACC.

| Dataset | Geometry (resolution mm3) | Timing & Params (times in ms) | flip angle of |
| --- | --- | --- | --- |
| ADNI | 1× 1 × 1 | TI=900 ms TE=min full TR=2300 | 9° |
| NACC | 1 × 1 × 1 - 1.2 × 1.2 × 1.2 | TI=400-1100ms  TE=3ms  TR=2300-3000 | 7°-11° |

Supplementary Table S1: Diagnosis Criteria for Cognitively Normal, Mild Cognitive Impairment and Mild Alzheimer’s Disease Dementia in two cohorts: ADNI and NACC.

| Criteria | ADNI | NACC |
| --- | --- | --- |
| Cognitively Normal | - No Memory Complaints aside from those common to other normal subjects of that age range. - Normal memory function documented by scoring at specific cutoffs on the Logical Memory II subscale (delayed Paragraph Recall) from the Wechsler Memory Scale - Revised (the maximum score is 25): a) greater than or equal to 9 for 16 or more years of education b) greater than or equal to 5 for 8-15 years of education c) greater than or equal to 3 for 0-7 years of education. - Mini-Mental State Exam score between 24 and 30 (inclusive) (Exceptions may be made for subjects with less than 8 years of education at the discretion of the project director). - Clinical Dementia Rating = 0. Memory Box score must be 0. - Cognitively normal, based on an absence of significant impairment in cognitive functions or activities of daily living. | The subject has normal cognition (global CDR=0 and/or neuropsychological testing within normal range) and normal behavior (i.e., the subject does not exhibit behavior sufficient to diagnose MCI or dementia due to FTLD or LBD) |
| MCI | - Memory complaint by subject or study partner that is verified by a study partner. - Abnormal memory function documented by scoring below the education adjusted cutoff on the Logical Memory II subscale (Delayed Paragraph Recall) from the Wechsler Memory Scale – Revised (the maximum score is 25): a) less than or equal to 8 for 16 or more years of education b) less than or equal to 4 for 8-15 years of education c) less than or equal to 2 for 0-7 years of education. - Mini-Mental State Exam score between 24 and 30 (inclusive) (Exceptions may be made for subjects with less than 8 years of education at the discretion of the project director). - Clinical Dementia Rating = 0.5. Memory Box score must be at least 0.5. - General cognition and functional performance sufficiently preserved such that a diagnosis of Alzheimer’s disease cannot be made by the site physician at the time of the screening visit. | If the subject does not have normal cognition or behavior and is not clinically demented, indicate the type of cognitive impairment below.  MCI CORE CLINICAL CRITERIA • Is the subject, the co-participant, or a clinician concerned about a change in cognition compared to the subject’s previous level?  •  Is there impairment in one or more cognitive domains (memory, language, executive function, attention, and visuospatial skills)?  •  Is there largely preserved independence in functional abilities (no change from prior manner of functioning or uses minimal aids or assistance)?  (Excluding any cases with NACCALZP=2, 3, or 7) |
| AD | - Memory complaint by subject or study partner that is verified by a study partner. - Abnormal memory function documented by scoring below the education adjusted cutoff on the Logical Memory II subscale (Delayed Paragraph Recall) from the Wechsler Memory Scale – Revised (the maximum score is 25): a) less than or equal to 8 for 16 or more years of education b) less than or equal to 4 for 8-15 years of education c) less than or equal to 2 for 0-7 years of education. - MMSE between 20 and 26 (inclusive) (Exceptions may be made for subjects with less than 8 years of education at the discretion of the protocol PI). - Clinical Dementia Rating = 0.5, 1.0 ➪ NINCDS/ADRDA criteria for probable AD. | The subject has cognitive or behavioral (neuropsychiatric) symptoms that meet all of the following criteria:   •  Interfere with ability to function as before at work or at usual activities? Represent a decline from previous levels of functioning?  • Are not explained by delirium or major psychiatric disorder?  •  Include cognitive impairment detected and diagnosed through a combination of 1) history-taking and 2) objective cognitive assessment (bedside or neuropsychological testing)?  AND Impairment in one* or more of the following domains.  – Impaired ability to acquire and remember new information  – Impaired reasoning and handling of complex tasks, poor judgment  – Impaired visuospatial abilities  – Impaired language functions  – Changes in personality, behavior, or comportment  * In the event of single-domain impairment (e.g., language in PPA, behavior in bvFTD, posterior cortical atrophy), the subject must not fulfill criteria for MCI.  (Excluding any cases with NACCALZP=2, 3, or 7) |

Supplementary Table S2: List of  138 volumes and thickness of  clinically-relevant brain ROIs, obtained by Freesurfer for each included subject.

| **Volumes or Thickness Variables Based on Brain ROIs** | | | |
| --- | --- | --- | --- |
| Left-Lateral-Ventricle | Right-WM-hypointensities | lh_entorhinal_volume | rh_cuneus_volume |
| Left-Inf-Lat-Vent | non-WM-hypointensities | lh_fusiform_volume | rh_entorhinal_volume |
| Left-Cerebellum-White-Matter | Left-non-WM-hypointensities | lh_inferiorparietal_volume | rh_fusiform_volume |
| Left-Cerebellum-Cortex | Right-non-WM-hypointensities | lh_inferiortemporal_volume | rh_inferiorparietal_volume |
| Left-Thalamus-Proper | Optic-Chiasm | lh_isthmuscingulate_volume | rh_inferiortemporal_volume |
| Left-Caudate | CC_Posterior | lh_lateraloccipital_volume | rh_isthmuscingulate_volume |
| Left-Putamen | CC_Mid_Posterior | lh_lateralorbitofrontal_volume | rh_lateraloccipital_volume |
| Left-Pallidum | CC_Central | lh_lingual_volume | rh_lateralorbitofrontal_volume |
| 3rd-Ventricle | CC_Mid_Anterior | lh_medialorbitofrontal_volume | rh_lingual_volume |
| 4th-Ventricle | CC_Anterior | lh_middletemporal_volume | rh_medialorbitofrontal_volume |
| Brain-Stem | BrainSegVol | lh_parahippocampal_volume | rh_middletemporal_volume |
| Left-Hippocampus | BrainSegVolNotVent | lh_paracentral_volume | rh_parahippocampal_volume |
| Left-Amygdala | BrainSegVolNotVentSurf | lh_parsopercularis_volume | rh_paracentral_volume |
| CSF | lhCortexVol | lh_parsorbitalis_volume | rh_parsopercularis_volume |
| Left-Accumbens-area | rhCortexVol | lh_parstriangularis_volume | rh_parsorbitalis_volume |
| Left-VentralDC | CortexVol | lh_pericalcarine_volume | rh_parstriangularis_volume |
| Left-vessel | lhCerebralWhiteMatterVol | lh_postcentral_volume | rh_pericalcarine_volume |
| Left-choroid-plexus | rhCerebralWhiteMatterVol | lh_posteriorcingulate_volume | rh_postcentral_volume |
| Right-Lateral-Ventricle | CerebralWhiteMatterVol | lh_precentral_volume | rh_posteriorcingulate_volume |
| Right-Inf-Lat-Vent | SubCortGrayVol | lh_precuneus_volume | rh_precentral_volume |
| Right-Cerebellum-White-Matter | TotalGrayVol | lh_rostralanteriorcingulate_volume | rh_precuneus_volume |
| Right-Cerebellum-Cortex | SupraTentorialVol | lh_rostralmiddlefrontal_volume | rh_rostralanteriorcingulate_volume |
| Right-Thalamus-Proper | SupraTentorialVolNotVent | lh_superiorfrontal_volume | rh_rostralmiddlefrontal_volume |
| Right-Caudate | SupraTentorialVolNotVentVox | lh_superiorparietal_volume | rh_superiorfrontal_volume |
| Right-Putamen | MaskVol | lh_superiortemporal_volume | rh_superiorparietal_volume |
| Right-Pallidum | BrainSegVol-to-eTIV | lh_supramarginal_volume | rh_superiortemporal_volume |
| Right-Hippocampus | MaskVol-to-eTIV | lh_frontalpole_volume | rh_supramarginal_volume |
| Right-Amygdala | lhSurfaceHoles | lh_temporalpole_volume | rh_frontalpole_volume |
| Right-Accumbens-area | rhSurfaceHoles | lh_transversetemporal_volume | rh_temporalpole_volume |
| Right-VentralDC | SurfaceHoles | lh_insula_volume | rh_transversetemporal_volume |
| Right-vessel | EstimatedTotalIntraCranialVol | BrainSegVolNotVent | rh_insula_volume |
| Right-choroid-plexus | lh_bankssts_volume | eTIV |  |
| 5th-Ventricle | lh_caudalanteriorcingulate_volume | rh_bankssts_volume |  |
| WM-hypointensities | lh_caudalmiddlefrontal_volume | rh_caudalanteriorcingulate_volume |  |
| Left-WM-hypointensities | lh_cuneus_volume | rh_caudalmiddlefrontal_volume |  |

Supplementary Table S3: Performance of deep learning model and the ROI-volume/thickness model on patient subgroups based on Gender, Education and ApoE4 status.

|  | ADNI Heldout Patients   (n=90 individuals, 297 scans) | | NACC external validation   (n=1345 individuals, 1845 scans  ) | |
| --- | --- | --- | --- | --- |
|  | Deep learning model  Area under ROC curve | ROI-volume/thickness  Area under ROC curve | Deep learning model  Area under ROC curve | ROI-volume/thickness  Area under ROC curve |
| **Gender: Male** |  |  |  |  |
| Cognitively Normal | 88.42  (95% CI: 87.51-89.34) | 84.95  (95% CI: 84.11-85.79) | 83.74 (95% CI: 83.25 - 84.22) | 82.97  (95% CI: 82.48-83.47) |
| Mild Cognitive Impairment | 57.59  (95% CI: 55.89-59.30) | 55.19  (95% CI: 53.71-56.66) | 60.05 (95% CI: 59.39 - 60.70) | 62.31 (95% CI: 61.43 - 63.19) |
| Alzheimer's disease Dementia | 90.62  (95% CI: 89.64-91.61) | 77.79  (95% CI: 77.04-78.55) | 87.56 (95% CI: 86.97 - 88.15) | 83.91 (95% CI: 83.39 - 84.43) |
| **Gender: Female** |  |  |  |  |
| Cognitively Normal | 92.85  (95% CI: 92.18-93.53) | 83.96  (95% CI: 83.21-84.72) | 82.46 (95% CI: 82.26 - 82.66) | 80.04 (95% CI: 79.76 - 80.33) |
| Mild Cognitive Impairment | 66.35  (95% CI: 64.53-68.16) | 63.85  (95% CI: 62.44-65.27) | 66.18 (95% CI: 65.43 - 66.93) | 58.12 (95% CI: 57.57 - 58.68) |
| Alzheimer's disease Dementia | 99.21  (95% CI: 98.70-99.71) | 89.90  (95% CI: 89.10-90.70) | 92.74 (95% CI: 92.27 - 93.21) | 85.85 (95% CI: 85.38 - 86.33) |
| **Education <15 Years** |  |  |  |  |
| Cognitively Normal | 94.86  (95% CI: 94.39-95.34) | 93.34  (95% CI: 92.58-94.10) | 83.75 (95% CI: 83.24 - 84.27) | 80.72 (95% CI: 80.20 - 81.23) |
| Mild Cognitive Impairment | 52.88  (95% CI: 50.74-55.02) | 66.70  (95% CI: 65.04-68.35) | 59.10 (95% CI: 58.47 - 59.73) | 61.23 (95% CI: 60.46 - 62.00) |
| Alzheimer's disease Dementia | 88.42  (95% CI: 87.38-89.46) | 86.90  (95% CI: 85.92-87.88) | 87.00 (95% CI: 86.47 - 87.54) | 84.63 (95% CI: 83.76 - 85.50) |
| **Education >=15 Years** |  |  |  |  |
| Cognitively Normal | 86.66  (95% CI: 85.75-87.58) | 82.25  (95% CI: 81.17-83.33) | 82.14 (95% CI: 81.91 - 82.37) | 82.58 (95% CI: 82.20 - 82.96) |
| Mild Cognitive Impairment | 63.64  (95% CI: 62.69-64.60) | 55.50  (95% CI: 54.04-56.95) | 65.30 (95% CI: 64.73 - 65.86) | 58.75 (95% CI: 57.88 - 59.63) |
| Alzheimer's disease Dementia | 92.73  (95% CI: 92.19-93.28) | 82.70  (95% CI: 82.02-83.38) | 91.02 (95% CI: 90.63 - 91.42) | 86.31 (95% CI: 85.66 - 86.96) |
| **With ApoE4** |  |  |  |  |
| Cognitively Normal | 63.82  (95% CI: 62.11-65.52) | 80.92  (95% CI: 79.80-82.05) | 81.55 (95% CI: 81.14 - 81.95) | 85.53 (95% CI: 85.17 - 85.88) |
| Mild Cognitive Impairment | 47.47  (95% CI: 46.61-48.32) | 64.19  (95% CI: 63.27-65.11) | 59.70 (95% CI: 59.03 - 60.37) | 63.55 (95% CI: 63.17 - 63.94) |
| Alzheimer's disease Dementia | 81.78  (95% CI: 81.03-82.54) | 75.16  (95% CI: 74.30-76.01) | 88.44 (95% CI: 88.09 - 88.79) | 86.70 (95% CI: 86.48 - 86.93) |
| **Without ApoE4** |  |  |  |  |
| Cognitively Normal | 82.37  (95% CI: 81.67-83.07) | 72.82  (95% CI: 72.31-73.33) | 84.82 (95% CI: 84.64 - 85.00) | 78.81 (95% CI: 78.59 - 79.04) |
| Mild Cognitive Impairment | 68.57  (95% CI: 67.59-69.56) | 52.88  (95% CI: 51.96-53.80) | 67.24 (95% CI: 66.81 - 67.67) | 56.36 (95% CI: 55.90 - 56.82) |
| Alzheimer's disease Dementia | 91.74  (95% CI: 90.82-92.65) | 81.18  (95% CI: 80.19-82.18) | 91.20 (95% CI: 90.91 - 91.48) | 83.70 (95% CI: 83.45 - 83.95) |

Supplementary Table S4: Importance of ROIs in deep learning and ROI-volume/thickness model in classification of CN/MCI/AD. Details of our approach to compute importances is in the methods section.

| **Rank** | **ROIs** | **CN**  **Normed Gradient Count** | **MCI**  **Normed Gradient Count** | **AD**  **Normed Gradient Count** | **ROI model Feature Impor.** | **Rank** | **ROIs** | **CN**  **Normalized Gradient Count** | **MCI**  **Normed Gradient Count** | **AD**  **Normed Gradient Count** | **ROI model Feature Import.** |
| --- | --- | --- | --- | --- | --- | --- | --- | --- | --- | --- | --- |
| 1 | **4th-Ventricle** | 12.55 | 8.23 | 13.23 | 0.02 | 52 | **rh_cuneus** | 7.97 | 2.25 | 7.18 | 0.00 |
| 2 | **Left-Hippocampus** | 9.93 | 2.33 | 11.86 | 0.12 | 53 | **Right-Accumbens-area** | 6.31 | 2.81 | 7.85 | 0.01 |
| 3 | **rh_parahippocampal** | 10.01 | 2.19 | 11.84 | 0.01 | 54 | **rh_lateralorbitofrontal** | 7.85 | 1.95 | 7.28 | 0.00 |
| 4 | **CSF** | 10.88 | 3.03 | 11.75 | 0.00 | 55 | **lh_temporalpole** | 7.67 | 0.81 | 7.84 | 0.01 |
| 5 | **rh_isthmuscingulate** | 10.22 | 4.04 | 11.67 | 0.01 | 56 | **rh_precentral** | 7.78 | 1.27 | 7.69 | 0.00 |
| 6 | **lh_parahippocampal** | 10.03 | 1.94 | 11.65 | 0.02 | 57 | **lh_medialorbitofrontal** | 7.60 | 1.87 | 7.30 | 0.00 |
| 7 | **rh_entorhinal** | 9.78 | 0.38 | 11.55 | 0.02 | 58 | **rh_superiortemporal** | 7.32 | 0.41 | 7.59 | 0.01 |
| 8 | **lh_entorhinal** | 10.12 | 0.60 | 11.54 | 0.02 | 59 | **Right-Pallidum** | 6.76 | 0.87 | 7.47 | 0.01 |
| 9 | **lh_transversetemporal** | 11.43 | 2.54 | 11.49 | 0.00 | 60 | **lh_pericalcarine** | 7.38 | 1.93 | 7.45 | 0.00 |
| 10 | **Right-Amygdala** | 9.03 | 2.99 | 11.40 | 0.01 | 61 | **Left-choroid-plexus** | 7.31 | 0.93 | 7.34 | 0.00 |
| 11 | **Right-Hippocampus** | 9.38 | 1.31 | 11.28 | 0.03 | 62 | **Right-Lateral-Ventricle** | 7.33 | 3.00 | 6.38 | 0.01 |
| 12 | **WM-hypointensities** | 11.12 | 3.22 | 9.48 | 0.03 | 63 | **rh_supramarginal** | 6.99 | 1.51 | 7.21 | 0.00 |
| 13 | **rh_transversetemporal** | 10.24 | 1.36 | 11.07 | 0.00 | 64 | **lh_postcentral** | 7.18 | 1.55 | 6.91 | 0.00 |
| 14 | **rh_posteriorcingulate** | 10.89 | 2.07 | 11.06 | 0.00 | 65 | **rh_postcentral** | 6.92 | 1.10 | 7.17 | 0.01 |
| 15 | **lh_isthmuscingulate** | 9.93 | 4.18 | 11.00 | 0.01 | 66 | **lh_cuneus** | 7.14 | 1.99 | 6.84 | 0.02 |
| 16 | **lh_insula** | 10.38 | 1.92 | 10.71 | 0.00 | 67 | **Left-Lateral-Ventricle** | 7.14 | 0.99 | 6.30 | 0.01 |
| 17 | **CC_Posterior** | 9.57 | 1.88 | 10.42 | 0.01 | 68 | **lh_rostralanteriorcingulate** | 7.03 | 2.10 | 5.77 | 0.01 |
| 18 | **Left-Amygdala** | 8.25 | 3.45 | 10.42 | 0.07 | 69 | **rh_caudalmiddlefrontal** | 6.97 | 0.81 | 6.54 | 0.00 |
| 19 | **lh_posteriorcingulate** | 9.59 | 2.66 | 10.30 | 0.00 | 70 | **lh_lateralorbitofrontal** | 6.91 | 1.42 | 6.08 | 0.00 |
| 20 | **rh_insula** | 9.70 | 1.51 | 10.29 | 0.00 | 71 | **Left-Pallidum** | 6.79 | 1.58 | 6.91 | 0.01 |
| 21 | **Right-VentralDC** | 9.30 | 1.35 | 10.07 | 0.00 | 72 | **lh_superiortemporal** | 6.90 | 0.58 | 6.79 | 0.01 |
| 22 | **rh_precuneus** | 9.73 | 2.89 | 10.05 | 0.02 | 73 | **lh_inferiortemporal** | 6.29 | 0.42 | 6.72 | 0.00 |
| 23 | **rh_caudalanteriorcingulate** | 9.80 | 0.82 | 9.08 | 0.01 | 74 | **lh_superiorfrontal** | 6.59 | 0.68 | 5.18 | 0.00 |
| 24 | **Right-Thalamus-Proper** | 9.01 | 1.78 | 9.76 | 0.00 | 75 | **rh_medialorbitofrontal** | 6.52 | 2.20 | 5.99 | 0.00 |
| 25 | **rh_bankssts** | 9.10 | 1.41 | 9.72 | 0.01 | 76 | **rh_superiorfrontal** | 6.41 | 0.56 | 5.30 | 0.01 |
| 26 | **Left-Caudate** | 9.40 | 2.14 | 9.67 | 0.00 | 77 | **Right-choroid-plexus** | 6.37 | 3.05 | 5.96 | 0.00 |
| 27 | **rh_lingual** | 9.12 | 2.65 | 9.54 | 0.00 | 78 | **lh_superiorparietal** | 6.35 | 0.75 | 5.57 | 0.01 |
| 28 | **Left-VentralDC** | 8.69 | 1.62 | 9.52 | 0.00 | 79 | **lh_supramarginal** | 6.18 | 1.49 | 6.32 | 0.01 |
| 29 | **CC_Mid_Posterior** | 9.50 | 1.78 | 9.21 | 0.00 | 80 | **rh_superiorparietal** | 6.19 | 1.20 | 5.57 | 0.01 |
| 30 | **lh_fusiform** | 8.42 | 1.28 | 9.47 | 0.01 | 81 | **rh_inferiortemporal** | 6.14 | 0.43 | 6.14 | 0.01 |
| 31 | **3rd-Ventricle** | 9.32 | 4.15 | 8.91 | 0.01 | 82 | **rh_middletemporal** | 6.05 | 0.51 | 6.12 | 0.02 |
| 32 | **Left-Thalamus-Proper** | 8.55 | 2.23 | 9.32 | 0.00 | 83 | **Left-Accumbens-area** | 5.11 | 1.45 | 6.02 | 0.00 |
| 33 | **lh_parsopercularis** | 9.30 | 2.19 | 8.53 | 0.01 | 84 | **rh_parstriangularis** | 5.93 | 0.58 | 5.50 | 0.01 |
| 34 | **rh_pericalcarine** | 9.29 | 3.14 | 9.26 | 0.00 | 85 | **Right-Cerebellum-Cortex** | 5.80 | 0.84 | 5.47 | 0.00 |
| 35 | **CC_Mid_Anterior** | 9.17 | 1.81 | 9.26 | 0.01 | 86 | **rh_inferiorparietal** | 5.58 | 1.71 | 5.72 | 0.00 |
| 36 | **Right-Caudate** | 9.20 | 2.45 | 9.04 | 0.00 | 87 | **lh_parstriangularis** | 5.69 | 0.91 | 4.00 | 0.02 |
| 37 | **rh_parsopercularis** | 9.03 | 1.52 | 9.15 | 0.00 | 88 | **Right-CerebellumWhitMtr** | 5.68 | 0.40 | 5.52 | 0.00 |
| 38 | **lh_precuneus** | 8.79 | 2.34 | 9.15 | 0.01 | 89 | **Left-Cerebellum-Cortex** | 5.38 | 0.90 | 5.57 | 0.00 |
| 39 | **lh_lingual** | 8.52 | 2.29 | 9.10 | 0.01 | 90 | **Left-Putamen** | 5.33 | 1.98 | 5.52 | 0.00 |
| 40 | **lh_caudalanteriorcingulate** | 8.99 | 1.27 | 8.47 | 0.01 | 91 | **lh_middletemporal** | 5.37 | 0.37 | 5.49 | 0.01 |
| 41 | **rh_paracentral** | 8.89 | 0.63 | 8.67 | 0.01 | 92 | **Left-CerebellumWhiteMTr** | 5.36 | 0.65 | 5.46 | 0.00 |
| 42 | **CC_Central** | 8.84 | 0.50 | 8.27 | 0.00 | 93 | **lh_inferiorparietal** | 5.13 | 0.83 | 5.30 | 0.01 |
| 43 | **CC_Anterior** | 8.77 | 4.72 | 8.41 | 0.01 | 94 | **Right-Putamen** | 4.96 | 1.14 | 5.27 | 0.00 |
| 44 | **lh_paracentral** | 8.44 | 0.96 | 8.47 | 0.01 | 95 | **rh_lateraloccipital** | 5.18 | 0.90 | 3.90 | 0.00 |
| 45 | **rh_rostralanteriorcingulate** | 8.45 | 2.28 | 6.99 | 0.02 | 96 | **rh_parsorbitalis** | 4.79 | 0.43 | 4.08 | 0.01 |
| 46 | **rh_fusiform** | 8.08 | 1.23 | 8.39 | 0.01 | 97 | **lh_rostralmiddlefrontal** | 4.68 | 0.46 | 3.30 | 0.00 |
| 47 | **lh_bankssts** | 8.31 | 2.08 | 8.02 | 0.01 | 98 | **lh_lateraloccipital** | 4.23 | 0.29 | 3.76 | 0.02 |
| 48 | **lh_caudalmiddlefrontal** | 8.29 | 1.20 | 6.78 | 0.01 | 99 | **bk/others** | 4.23 | 0.67 | 3.96 |  |
| 49 | **rh_temporalpole** | 7.92 | 0.87 | 8.24 | 0.00 | 100 | **rh_rostralmiddlefrontal** | 3.97 | 0.20 | 3.79 | 0.00 |
| 50 | **Brain-Stem** | 7.73 | 0.88 | 8.10 | 0.01 | 101 | **lh_parsorbitalis** | 3.57 | 0.11 | 2.84 | 0.00 |
| 51 | **lh_precentral** | 8.06 | 1.30 | 7.39 | 0.00 |  |  |  |  |  |  |

Supplementary Table S5: Confusion matrix of the deep learning model in classification of CN/MCI/AD on ADNI heldout set. Rows represent the diagnosed classes and columns represent predictions of the model.

|  | **CN** | **MCI** | **AD** |
| --- | --- | --- | --- |
| **CN** | 76 | 9 | 4 |
| **MCI** | 34 | 44 | 33 |
| **AD** | 4 | 7 | 86 |

Supplementary Table S6: Confusion matrix of the deep learning model in classification of CN/MCI/AD on NACC dataset. Rows represent the diagnosed classes and columns represent predictions of the model.

|  | **CN** | **MCI** | **AD** |
| --- | --- | --- | --- |
| **CN** | 669 | 458 | 154 |
| **MCI** | 55 | 121 | 146 |
| **AD** | 17 | 44 | 3 81 |

Supplementary Table S7: Comparison of the published models to our best proposed models. we use the same settings as our proposed model (data augmentations are Gaussian blurring with σ uniformly chosen from 0 to 1.5, and random cropping of size 96 × 96 × 96. The optimizer is SGD with the momentum equal to 0.9). However, we adopted a larger batch size which is set to 16 since these architectures use batch normalization. The Learning rate is chosen by grid search from [1e-4, 1e-3, 1e-2, 1e-1] based on validation set performance, and is set to 0.001 and the models are early stopped based on validation loss.

| **Method** | Accuracy | Micro-AUC | Macro-AUC |
| --- | --- | --- | --- |
| ResNet-18 [(Valliani and Soni2017)](https://paperpile.com/c/LqODlB/kQWO) | 50.8% | - | - |
| ResNet-18 3D  [(Fung et al. 2019)](https://paperpile.com/c/LqODlB/Jbmf) | 52.4% | - | - |
| ResNet-18 3D | 50.1 ± 1.1% | 71.2 ± 0.4% | 72.4 ± 0.7% |
| Ours | 68.2 ± 1.1% | 82.0 ± 0.2% | 80.0 ± 0.5% |

Supplementary Table S8: Comparison of batch normalization (BN) and instance normalization (IN) layers on ResNet-18 and our best proposed models.

| **Method** | Accuracy | Micro-AUC | Macro-AUC |
| --- | --- | --- | --- |
| ResNet-18 3D with Batch Norm | 50.1% ± 1.1% | 71.2% ± 0.4% | 72.4% ± 0.7% |
| ResNet-18 3D with Instance Norm | 52.3% ± 0.8% | 74.1 ± 0.7% | 73.1% ± 0.9% |
| Proposed model with Batch Norm | 58.8% ± 0.9% | 75.9 ± 0.7% | 73.1 ± 0.8% |
| Proposed model with Instance Norm | 66.9% ± 1.2% | 82.0 ± 0.7% | 78.5 ± 0.7% |

**ADNI                                                                 NACC**

**
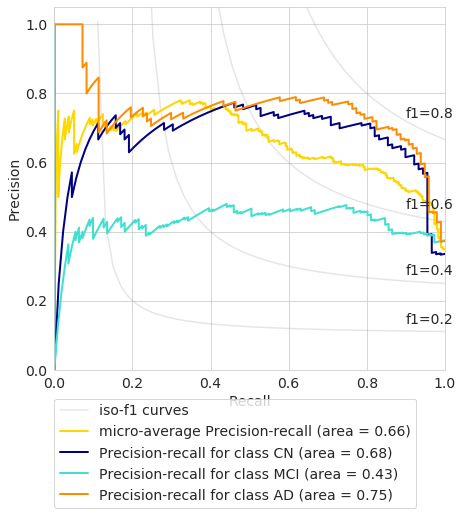
   
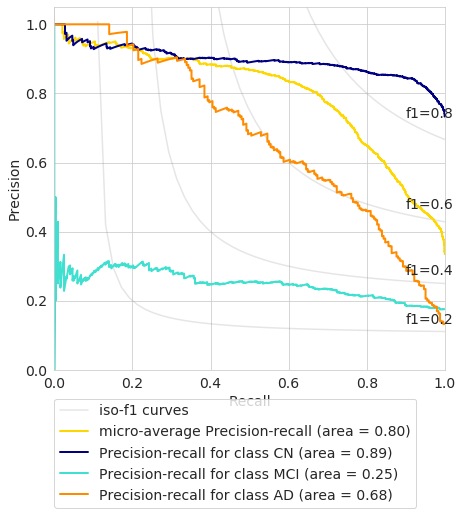
**

Supplementary Figure F1: Precision Recall Curve for prediction of CN (Class 0), MCI (Class 1) and AD (Class 2) in ADNI heldout test set and NACC external validation cohorts.


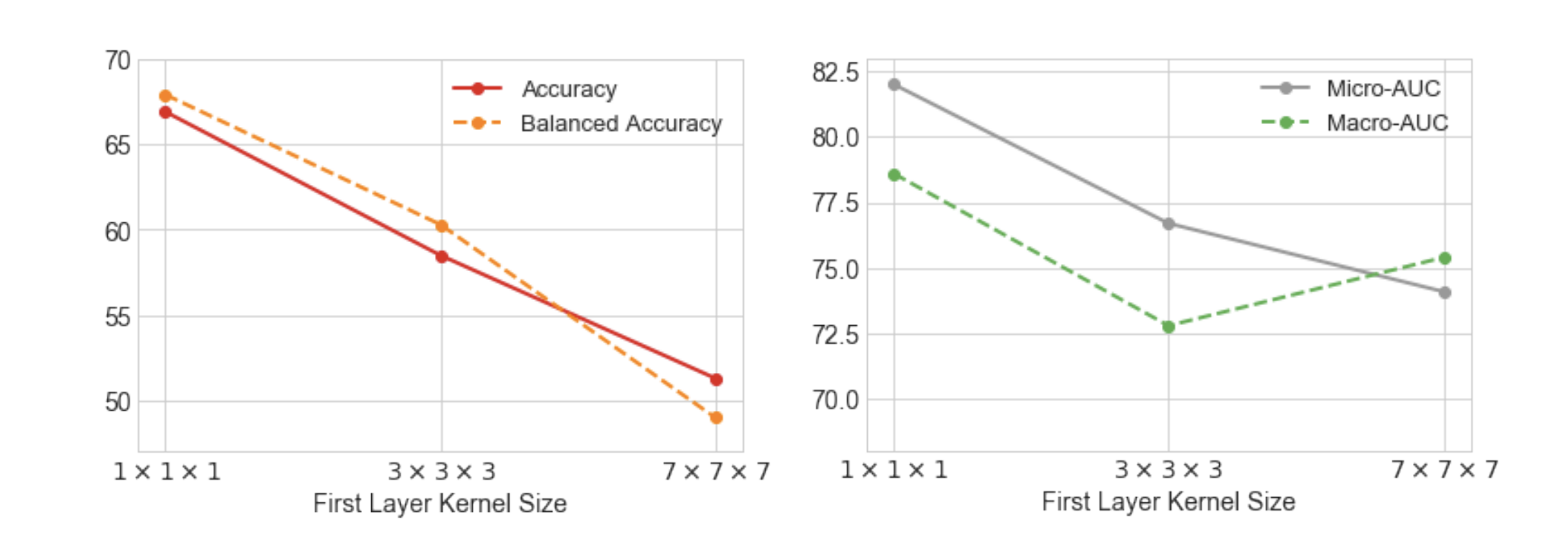


Supplementary Figure F2: Comparison of the performance of different first layer kernel sizes for both the proposed architecture.
